# When bacteriophages encounter macrophages during their journey through the human body

**DOI:** 10.3389/fcimb.2026.1857968

**Published:** 2026-05-28

**Authors:** Martyna Cieślik, Magdalena Gębicka, Michał Wójcicki, Andrzej Górski, Ewa Jończyk-Matysiak

**Affiliations:** 1 Bacteriophage Laboratory, Department of Phage Therapy, Hirszfeld Institute of Immunology and Experimental Therapy, Polish Academy of Sciences, Wroclaw, Poland; 2 Institute of Electronics, AGH University of Krakow, Krakow, Poland; 3 Department of Clinical Immunology, Medical University of Warsaw, Warsaw, Poland

**Keywords:** antibiotic resistance, bacteriophage, immunomodulation, macrophage, mononuclear phagocyte system, phagocytosis

## Abstract

The increasing problem of antibiotic resistance is one of the most significant threats to global public health, contributing to higher mortality rates, prolonged hospitalizations, and rising healthcare costs. As a response to this challenge, phage therapy – based on the use of bacteriophages (phages), viruses that selectively infect and lyse bacteria – has emerged as a promising alternative. In recent years, increasing interest in phage therapy has been observed in both preclinical and clinical research. Accordingly, increasing attention is being directed toward investigating potential interactions between phages and mammalian cells. Bacteriophages, like other exogenous particles introduced into the human body, are subject to innate and adaptive immune mechanisms. This review places particular emphasis on interactions between phages and macrophages. On the one hand, their significant participation in the clearance of therapeutic phages is described, which may reduce the effectiveness of antibacterial treatment; nevertheless, this mechanism is considered to be an indirect way of autotolerance of intestinal phages. From another perspective, bacteriophages, as nucleoprotein molecules, can induce various immunomodulation mechanisms, which may find clinical applications in the future. Their involvement in macrophage polarization, induction of the secretion of pro- and anti-inflammatory cytokines, and modulation of phagocytic functions are briefly discussed. Apart from their therapeutic importance, studying the interactions of phages with eukaryotic cells, such as immunocompetent cells including macrophages, may also help explain their indisputable role in maintaining homeostasis as integral components of the human microbiota.

## 1 Phage therapy during the antibiotic crisis

The growing phenomenon of antibiotic resistance represents one of the most serious threats to public health in the 21st century, leading to increased mortality, prolonged hospitalizations, and rising healthcare costs (Ahmad et al., 2023; Ajekiigbe et al., 2025; Patra et al., 2025; Wójcicki et al., 2026). Of particular concern is the increasing prevalence of multidrug-resistant strains, including pathogens belonging to the ESKAPE group, such as *Enterococcus faecium*, *Staphylococcus aureus*, *Klebsiella pneumoniae*, *Acinetobacter baumannii*, *Pseudomonas aeruginosa*, and *Enterobacter* spp (Cieślik et al., 2025; Woh and Zhang, 2025; Bajrak et al., 2026). According to the World Health Organization (WHO), drug-resistant bacterial infections are responsible for approximately 700,000 deaths annually, and this number is estimated to rise to 10 million by 2050 if effective therapeutic alternatives are not implemented (GBD Antimicrobial Resistance Collaborators, 2024). In 2024, the WHO published an updated list of drug-resistant bacteria posing the greatest threat to human health, aimed at guiding the development of new treatment strategies and measures to prevent and limit the spread of antimicrobial resistance (World Health Organization, 2025). Therefore, in the face of limited development of new antimicrobial agents and the rapid pace at which bacteria acquire resistance, the search for alternative therapeutic strategies has become imperative.

Phage therapy, which utilizes bacteriophages (phages) – viruses capable of specifically infecting and lysing bacteria – represents a promising solution to this problem (Hibstu et al., 2022; Oromí-Bosch et al., 2023; De et al., 2026). Bacteriophages exhibit two principal infection cycles – the lytic cycle, which leads to bacterial cell destruction and is particularly relevant for therapeutic applications, and the lysogenic cycle, in which the phage genome integrates into the host genome and replicates without immediate host cell lysis. Phages exhibit high host specificity, allowing selective elimination of bacterial pathogens while preserving the physiological microbiota of the host (El Haddad et al., 2022; Wójcicki et al., 2025a). Unlike broad-spectrum antibiotics, phage therapy minimizes the risk of dysbiosis and the development of secondary opportunistic infections, such as *Clostridioides difficile* infections (Venhorst et al., 2022; Raeisi et al., 2023; Mohiuddin, 2026). An additional advantage of bacteriophages is their ability to amplify at the site of infection. As a result, therapeutic efficacy may increase in the presence of target bacteria while declining following pathogen elimination, thereby reducing potential adverse effects (Peng et al., 2024; Wójcicki et al., 2025a). Furthermore, bacteriophages demonstrate the ability to penetrate and degrade bacterial biofilms using phage-encoded enzymes, such as depolymerases and endolysins, which represent a significant clinical challenge in chronic infections, implant-associated infections, and hospital-acquired infections (Amankwah et al., 2022; Cieślik et al., 2025; Scalia and Najmi, 2025; Wójcicki et al., 2025b). Studies have demonstrated that bacteriophages may act synergistically with antibiotics, enhancing treatment efficacy while reducing the risk of resistance development (Afrasiabi et al., 2025; Gliźniewicz et al., 2025). Moreover, the emergence of bacterial resistance to bacteriophages is frequently associated with reduced bacterial virulence or restoration of antibiotic susceptibility, providing an additional therapeutic benefit (Mangalea and Duerkop, 2020).

In recent years, increasing interest in phage therapy has been observed in both preclinical and clinical research, accompanied by a growing number of reported cases of its use in the treatment of infections resistant to conventional treatments (Melo et al., 2020; Pires et al., 2020; Gomez-Ochoa et al., 2022). Advances in genomic technologies, synthetic biology, and bacteriophage engineering enable the design of phages with enhanced efficacy, expanded host range, and reduced risk of transferring resistance genes (Lenneman et al., 2021; Attar, 2025). Consequently, phage therapy is currently regarded as one of the most promising tools in combating the global antibiotic resistance crisis and as an important component of future personalized medicine (Bhale et al., 2026).

## 2 The constant cooperation of phages with mammalian cells

Advances in modern large-scale sequencing technologies have enabled human microbiome analyses to generate an increasing amount of data on vast numbers of previously unexplored phages, both temperate and lytic (Dahlman et al., 2025; Miller-Ensminger et al., 2018; Sausset et al., 2020; Townsend et al., 2021). At the same time, the association between the composition of the human phageome and numerous diseases is becoming increasingly evident (Rybicka and Kaźmierczak, 2025; Vives, 2025; Wheatley et al., 2025). Bacteriophages, an integral component of the human microbiota, constantly interact with eukaryotic cells. The dynamics of phage-bacterium-mammalian cell interactions are currently one of the most important aspect in phage biology research. For example, it is believed that phages diffusing in the mucosal layer, due to the interactions with mucins, may protect further cells and tissues from bacterial invasion, as described on the example of model phage T4, *Escherichia coli*-specific phage ΦPNJ-9, and *Pseudomonas*-specific phages (especially GEC_MRC and GEC_PNG3) (Barr et al., 2013, 2015; Almeida et al., 2024; Fu et al., 2025). Some current research focuses on engineering of phages to improve their adhesion to epithelial cells (Grigonyte et al., 2021).

Interactions of naturally occurred bacteriophages (like T4 phage) or their proteins with immunocompetent cells, such as dendritic cells (Bocian et al., 2016; Miernikiewicz et al., 2013), have been repeatedly described. However, it should be noted that these effects were usually only slightly pronounced, which highlights the adaptation of mammalian cells to coexist with bacterial viruses. Recent studies directly demonstrating phage entry into mammalian cells have shown that, among the different cell types studied (endothelial, epithelial, fibroblasts, and phagocytic cells), T4 phage internalization by monocyte-induced macrophages was relatively weak (Bichet et al., 2021). On the other hand, the participation of virulent intestinal phages isolated from various animals in maintaining natural immunity against bacterial lipopolysaccharide (LPS) through appropriate programming of macrophages has been described (Hou et al., 2025). Interestingly, using the example of model T4 phage, it has been shown that some types of epithelial cells can endocytose phages and use them as a resource for improving growth, without inducing pro-inflammatory mechanisms (Bichet et al., 2023). The converse scenario may occur when therapeutic bacteriophages that do not constitute stable components of the human microbiome are administered. Numerous studies suggest that mammalian cell responses to lytic phages are heterogeneous. For example, lytic *Pseudomonas*-specific phages induced pro-inflammatory responses in the respiratory epithelium (Zamora et al., 2024). Studies in chickens also suggest activation of the pro-inflammatory Toll-like receptor 9 (TLR-9)-dependent pathway following administration of phages targeting *Salmonella* (Podlacha et al., 2024). In contrast, transcytosed phages are considered to not disrupt epithelial barrier integrity, as described for intestinal epithelia (Carroll-Portillo et al., 2025; Cieślik et al., 2026; Douadi et al., 2026; Nguyen et al., 2017; Wójcicki et al., 2025c) or respiratory tract epithelia (Zamora et al., 2024). Our findings further demonstrate that therapeutic *Enterobacter*-specific phages do not compromise the viability of intestinal epithelial cells and may even enhance it (Cieślik et al., 2026). Freyberger et al. (2018) also described the lack of significant induction of an immune response by monocytes after phage administration.

**Figure 1:**
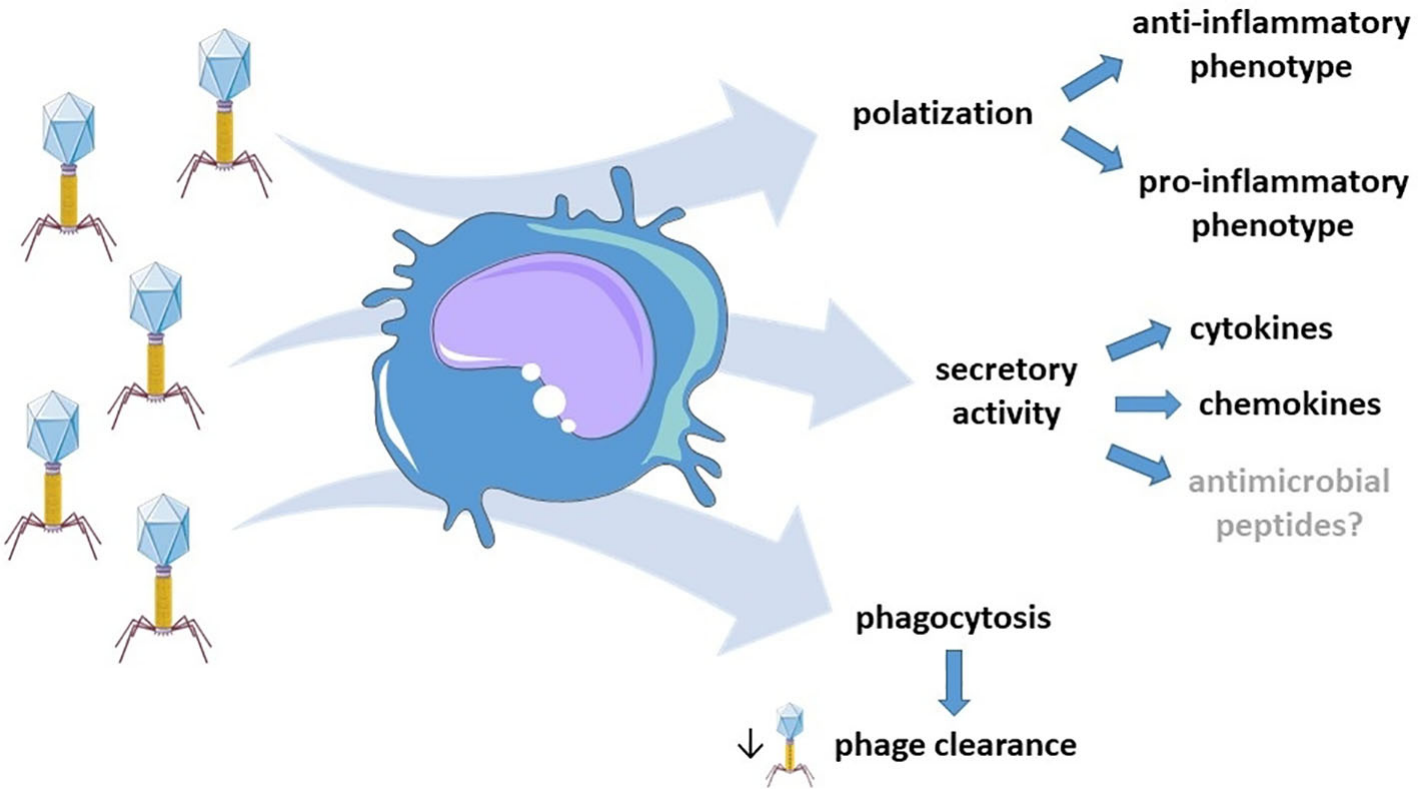
Potential effects of bacteriophages on macrophages. Bacteriophages, as nucleoprotein particles, can be recognized by immune cells, including macrophages. Phagocytosis of bacteriophages can lead to their increased clearance from the body. However, phages can modulate macrophage functions, such as the secretion of pro- and anti-inflammatory cytokines and chemokines, and can further influence their polarization. Certain images were provided by Servier Medical Art (https://smart.servier.com), licensed under CC BY 4.0.

In light of the above, the influence of bacteriophages on mammalian cells is undeniable (Górski et al., 2017). The remaining question, however, is whether these interactions can be harnessed to our advantage (**Figure 1**). In this review, we provide a comprehensive and in-depth examination of the exploitable functional capabilities of macrophages following their possible contact with potentially therapeutic bacteriophages.

## 3 The role of macrophages in the bacteriophage clearance

Bacteriophages, like other exogenous particles introduced into the human body, are subject to innate immune mechanisms that may lead to their neutralization. It is known that the development of immune response after phage administration may increase their clearance (Berkson et al., 2024), significantly influencing phage pharmacokinetics and pharmacodynamics. Nevertheless, the role of neutrophils – the most common phagocytic cells in the body – in the clearance of administered phages is very limited (Echterhof et al., 2024). On the other side, the significant role of phagocytic neutrophils in supporting phage therapy for *P. aeruginosa* pulmonary infection has recently been described (Weissfuss et al., 2025a). Conversely, monocytes can engulf and phagocytose phages in a time- and dose-dependent manner (Perea et al., 2021). The important role of both liver sinusoidal endothelial cells and Kupffer cells in the uptake of gut-derived or intravenous administered phages from circulation and their further degradation was also noted (Øie et al., 2020; Sánchez Romano et al., 2024). This mechanism can be considered a type of immunological tolerance to phages that are a natural component of the human microbiome. Although the concept of immune tolerance to bacteriophages has been proposed, especially for resident phages, experimental evidence remains limited. Current reports indicate that repeated stimulation with bacteria-derived temperate phages may induce cross-reactive antobodies further neutralizing therapeutic phages (Gordillo Altamirano et al., 2026). Moreover, therapeutic phage administration may more frequently induce anti-phage immune responses, including neutralizing antibodies and increased phage clearance and/or limit their antibacterial properties, which may reduce therapeutic efficacy (Bernabéu-Gimeno et al., 2024; Majewska et al., 2019). However, strong evidence is emerging that macrophages can also significantly influence the clearance of therapeutic bacteriophages. Phage particles can be internalized and ingested by macrophages, in particular, with splenic and liver macrophages playing a particularly important role in this process (Kaźmierczak et al., 2014; Hodyra-Stefaniak et al., 2015; Van Belleghem et al., 2018). Nevertheless, the total splenic macrophage population may not change in response to intraperitoneally administered phages (Berkson et al., 2024). Another example was described in a comparative model between alveolar macrophage-depleted mice versus immunocompetent ones. The model focused on infection with *P. aeruginosa* and phage treatment. Significantly more effective treatment outcomes were observed in the animals with depletion of macrophages, which, after further confirmation, indicated phagocytosis of administered phages by macrophages. Thus, excessive phage clearance by macrophages can reduce phage density and limit therapeutic efficacy (Zborowsky et al., 2025). Nevertheless, *Pseudomonas*-specific phages may be effective in lung infections in patients suffered from cystic fibrosis (Chan et al., 2025) or may successfully support antibiotic treatment of ventilator-associated-pneumonia (Weissfuss et al., 2025b).

Interestingly, in studies on the possibility of using nanotubes from the phage tail sheath protein as a novel drug transportation system, it turned out that these nanotubes are actively and passively internalized by peritoneal macrophages, simultaneously reducing the desirable therapeutic effect (Gabrielaitis et al., 2023).

From a phage therapy perspective, it is crucial to understand the pathways that will enable therapeutic phages to evade clearance by the immune system to remain in circulation and kill bacteria effectively (Le et al., 2025). It is worth noting that macrophage-mediated phage removal is one of several innate immune mechanisms limiting phage therapy, alongside, e.g., their complement-mediated inactivation (Egido et al., 2023), or those associated with acquired immunity, like phage neutralization by anti-phage antibodies (Berkson et al., 2024).

## 4 Targeted use of the immunomodulatory properties of bacteriophages

The mononuclear phagocyte system (MPS), of which tissue-resident macrophages are an important component, is considered a dispersed organ in the human body that performs many important functions maintaining homeostasis (Gordon and Plüddemann, 2019). Phenotypic or genotypic changes in macrophages may also play a significant role in the pathogenesis of various disorders. One of these are autoimmune diseases, which can be classified as systemic (e.g., systemic lupus erythematosus) or organ-specific (e.g., type 1 diabetes). Although their etiology is highly diverse and their pathogenesis remains incompletely understood, these disorders share several common features, including activation of the innate immune system, the presence of autoreactive antibodies, and T-cell responses directed against self-antigens (Yang et al., 2023). Examples of diseases in which macrophages play a crucial role are presented in **Table 1**.

**Table 1:** Examples of diseases in which macrophages play a significant role.

Disease	The role of macrophages	References
Intestinal inflammation (e.g., Crohn’s disease, ulcerative colitis)	The accumulation of macrophages with an inflammatory CD14^hi^ phenotype in the intestinal mucosa, which further exacerbates inflammation and damage of intestinal mucosa. Cytokines secreted by inflammatory-activated macrophages also contribute to tumorigenesis.	(Han et al., 2021; Zhang et al., 2023)
Systemic lupus erythematosus (SLE)	Macrophages polarized toward a pro-inflammatory state and the impaired efferocytosis mechanisms further lead to the accumulation of apoptotic material and the release of autoantigens, thereby amplifying inflammation and tissue damage.	(Ma et al., 2019; Brancewicz et al., 2025)
Rheumatoid arthritis (RA)	After crossing the synovial membrane, M1 macrophages stimulate synovial fibroblasts by secreting pro−inflammatory cytokines (e.g., TNF-α, IL-6), which promotes cartilage and bone degradation. In contrast, the persistent tissue−repair attempts driven by M2 macrophages contribute to pannus formation and fibrosis.	(Yang et al., 2020; Brancewicz et al., 2025)
Acute respiratory distress syndrome (ARDS)	Upon infection, macrophages undergo M1 polarization, triggering an inflammatory response that contributes to lung tissue damage. During later stages of infection, M2 macrophages participate in tissue repair; however, in advanced ARDS, excessive and prolonged M2 polarization may promote the development of pulmonary fibrosis.	(Tao et al., 2023)
Hashimoto’s thyroiditis	Macrophages that infiltrate the thyroid gland contribute to its damage by presenting antigens and secreting cytokines (e.g., IFN-γ), which promotes a Th1-polarization and leads to the recruitment of cytotoxic T lymphocytes targeting thyroid follicular cells.	(Wrońska et al., 2024; Brancewicz et al., 2025)

Given the involvement of macrophages in the pathogenesis of numerous diseases, we discuss their potential interactions with bacteriophages, with the aim of assessing their possible future utility as adjuncts to antimicrobial therapy or as targeted modulators in inflammatory diseases.

### 4.1 Polarization of macrophages

Macrophages play a crucial role in the innate immune response, contributing to the progression of inflammation, as well as to tissue repair and the maintenance of homeostasis. Under the influence of various environmental signals, macrophages polarize into different phenotypic states, within a broad activation spectrum, with the M1 and M2 phenotypes positioned at opposite ends of this continuum (Brancewicz et al., 2025). M1 macrophages are characterized as strongly pro-inflammatory cells with high phagocytic and cytotoxic activity, and the cytokines (e.g., IL-6, IL-12, TNF-α) and chemokines they produce play a key role in initiating and sustaining inflammatory responses, which, when excessive or prolonged, may contribute to the development of inflammatory and autoimmune diseases. In contrast, M2 macrophages exhibit anti−inflammatory functions and contribute to tissue repair and remodeling through the secretion of cytokines such as IL-10 and TGF-β (Brancewicz et al., 2025; Ross et al., 2021).

Macrophage stimulation with several virulent gut-derived phages (i.e., *S. aureus*-specific phage JS25; *Streptococcus pyogenes*-specific phage DELF; *P. aeruginosa*-specific phage 065; *E. coli*-specific phage DELF2; and ISF003 phage specific to *Shigella dysenteriae*) induces M1-like polarization, and, moreover, can switch M2 state towards M1 type polarization indicating their reprogramming capabilities (Hou et al., 2025). Induction of M1 macrophages may indicate stimulation of the immune system in response to bacterial infection to enhance the antibacterial response of phagocytic cells. On the other hand, phage-primed macrophages exhibited tolerance for subsequent stimulation with LPS, reducing levels of pro-inflammatory cytokines and building immune memory (Hou et al., 2025). In response to LPS administration, naive mice demonstrated hyperinflammation leading to death. In turn, phage stimulation induced the development of immune tolerance, maintaining immune memory (Hou et al., 2025). Interesting observations were noted with mouse peritoneal macrophages, which, when cultured on filamentous M13 phage-coated surfaces, showed significant polarization towards the M2 type (Safari et al., 2022). Similarly, recent reports have described the significant contribution of *E. cloacae*-specific phage vB_Ent31 to M2 macrophage polarization, which helped to suppress inflammation and enhance the healing of infected wound (Li et al., 2025).

### 4.2 Cytokine secretion

Another key function of macrophages is the secretion of a wide range of cytokines and chemokines that regulate immune responses. Through this activity, macrophages orchestrate inflammation, cell recruitment, and communication between innate and adaptive immune systems.

Interestingly, murine macrophages cultured on surface coated with M13 bacteriophage were promoted to the production of anti-inflammatory cytokines (IL-10 and TGF-β) with simultaneously decreasing levels of pro-inflammatory cytokines (TNF-α and IL-6; despite the initial increase), both under basal conditions and following LPS stimulation (Safari et al., 2022). In turn, *S. aureus*-specific phage phiMR003 did not induce the secretion of any cytokines by unstimulated peritoneal macrophages, but influence on a significant reduction in the pro-inflammatory IL-1β caused by exposure of cells to LPS (Suda et al., 2022). Conversely, two newly-isolated coliphages (P1 and P2) were able to stimulate the secretory activity of non-LPS-stimulated macrophages (Yıldızlı et al., 2020). In another study, phage JEP7 specific to *E. coli* stimulated TNF-α, IL-6, and IL-10 secretion, while *Bacillus cereus*-specific phage PBC2 induced IL-10 by macrophages *in vitro*, even though both phages did not differ in internalization by cells or cytotoxicity effect (Jung et al., 2025).

In the study described above, phage-induced, M1-polarized macrophages increased TNF-α secretion (Hou et al., 2025). However, in a murine model, after LPS stimulation, macrophages derived from phage-exposed mice showed reduced secretion of pro-inflammatory cytokines (i.e., TNF-α and IL-6) compared to those from unpretreated mice (Hou et al., 2025). In was recently described that directly applied T4 phage stimulated TNF-α production by macrophages in a concentration-dependent manner. Moreover, the purified genetic material of the T4 phage did not cause such an effect (Carroll-Portillo et al., 2025). Interestingly, the process of transcytosis through the epithelia may influence the enhancement of the immunomodulatory properties of bacteriophages. This phenomenon was recently demonstrated by the example of stronger induction of TNF-α secretion by macrophages after their stimulation with transcytosed T4 phage, compared to naive phage (Carroll-Portillo et al., 2025). Although the process of phage transcytosis/translocation through epithelia is considered harmless to them, the described phenomenon opens a completely new field of research on the influence of human body environment on the properties of phages residing in them and/or administered therapeutically. Apart from that, bacteriophages can also modulate epithelial functions, as demonstrated, among others, by the observation of increased gene expression for human defensins by intestinal epithelial cells after their stimulation with model T4 and *S. aureus*-specific A5/80 phages (Borysowski et al., 2020).

### 4.3 Phagocytic activity

Phagocytosis is a process aimed at eliminating particles larger than 0.5 μm, playing a key role in immune defense and tissue homeostasis (Uribe-Querol and Rosales, 2020). Macrophages participate actively in this process, migrating to sites of tissue damage in response to chemokines such as CCL2 and CX3CL1 during infection and inflammation (Guan et al., 2025). They phagocytose pathogens, apoptotic cells, and tissue debris, which are subsequently degraded in phagolysosomes, and they can internalize antigens for presentation to T and B cells, initiating adaptive immune responses.

Interactions between phages and phagocytes can be bidirectional. From one perspective, phages can enhance bacterial phagocytosis by acting as opsonins, making phage-coated bacteria more recognizable to the immune system and facilitating their elimination *in vivo* (Kaur et al., 2014; Urban-Chmiel and Pyzik, 2025). Recent studies have shown that marking the surface of bacteria with residual phage capsid accompanied with manganese ions leads to the formation of the artificial sites recognized by macrophages, consequently enabling phagocytosis via mannose receptors with the participation of the release of ions with bactericidal effect (Yao et al., 2025). This evidence supports the development of novel phage-based applications, including therapies targeting intracellular pathogens such as *Mycobacterium abscessus* surviving in phagosomes inside macrophages (Schmalstig et al., 2023; Kapoor et al., 2025) and methods for tracking phage distribution *in vivo* (Jończyk-Matysiak et al., 2017). However, interactions of phage particles with phagocytes may limit their therapeutic effectiveness, and this effect appears to be phage-specific, potentially influencing treatment outcomes. For example, a *P. aeruginosa* strain harboring the filamentous Pf4 phage shows reduced susceptibility to macrophage phagocytosis compared to strains lacking the phage (Secor et al., 2017; Sweere et al., 2019). In experimental model, splenic macrophages from mice with acute urinary tract infections displayed reduced intracellular bacterial killing compared to healthy controls (Jończyk-Matysiak, 2015). However, treatment with specific phage lysates enhanced macrophage bactericidal activity, allowing phagocytes to continue eliminating bacteria even after phagocytosis. Similarly, in patients with urinary tract infections, peripheral blood mononuclear cells (PBMCs) showed a significant increase in intracellular killing of the non-pathogenic *E. coli* strain B following phage therapy (Jończyk-Matysiak et al., 2015). These findings highlight the importance of phage-immune cell interactions in improving phage therapy outcomes. Nevertheless, interactions between phages, phagocytes, and bacteria are influenced by multiple factors. Notably, it has been demonstrated that exposure of murine macrophages to M13 phage reduces the phagocytic uptake of yeast cells, while concurrently enhancing the efferocytosis of apoptotic cells (Safari et al., 2022). These findings further support the concept that bacteriophages may play a significant indirect role in maintaining homeostasis.

## 5 Conclusions

As mentioned above, bacteriophages may induce weak or no immunological responses, which may be beneficial from the perspective of phage therapy efficacy. From another point of view, discovering the potential impact of naturally occurring bacteriophages in maintaining immune homeostasis is currently one of the most important directions in phage biology research. The composition of the phage component of the natural microbiome has been linked to multiple diseases, which may, in part, be explained by direct effects of specific phages on human cells. Moreover, understanding the precise interactions of bacteriophages with phagocytic cells may provide data necessary to explain the bio-distribution processes of therapeutic phages in the human body. Although phagocytosis of bacteriophages may limit their systemic distribution, their engulfment by professional phagocytes can simultaneously trigger immune responses that may be harnessed to influence therapeutic outcomes. It is important to highlight existing knowledge gaps, particularly whether phages can influence the secretion of antimicrobial peptides by macrophages (**Figure 1**), thereby potentially enhancing the treatment of infections. In addition, further studies on phage-mediated immunomodulation (including phage proteins) may provide novel means of therapy beyond phage-dependent antibacterial effects. Moreover, elucidating receptor-level interactions between bacteriophages and eukaryotic cells remains a major challenge and an important direction for future research.

In general, studies involving larger and more diverse sets of bacteriophages are still needed to determine which interactions are universal across phages and which are specific to particular groups or individual viruses.
